# Advances in Biodegradable Food Packaging Using Wheat-Based Materials: Fabrications and Innovations, Applications, Potentials, and Challenges

**DOI:** 10.3390/foods13182964

**Published:** 2024-09-19

**Authors:** Ravshanbek S. Alibekov, Klara U. Urazbayeva, Abdugani M. Azimov, Azri Shahir Rozman, Norhashila Hashim, Bernard Maringgal

**Affiliations:** 1 Food Biotechnology Scientific-Research Laboratory, M. Auezov’ South-Kazakhstan University, Tauke Khan Avenie, 5, Shymkent 160000, Kazakhstan; ralibekov@hotmail.com (R.S.A.); klara_abdrazak@mail.ru (K.U.U.); azimov-78@mail.ru (A.M.A.); 2 Department of Biological and Agricultural Engineering, Faculty of Engineering, Universiti Putra Malaysia, Serdang 43400, Selangor, Malaysia; azri.shahir@gmail.com; 3 SMART Farming Technology Research Centre (SFTRC), Faculty of Engineering, Universiti Putra Malaysia, Serdang 43400, Selangor, Malaysia; 4 Faculty of Resource Science and Technology, Universiti Malaysia Sarawak, Kota Samarahan 94300, Sarawak, Malaysia

**Keywords:** wheat, starch, gluten, fiber, innovation

## Abstract

This article explores the advancements in biodegradable food packaging materials derived from wheat. Wheat, a predominant global cereal crop, offers a sustainable alternative to conventional single-use plastics through its starch, gluten, and fiber components. This study highlights the fabrication processes of wheat-based materials, including solvent casting and extrusion, and their applications in enhancing the shelf life and quality of packaged foods. Recent innovations demonstrate effectiveness in maintaining food quality, controlling moisture content, and providing microbiological protection. Despite the promising potential, challenges such as moisture content and interfacial adhesion in composites remain. This review concludes with an emphasis on the environmental benefits and future trends in wheat-based packaging materials.

## 1. Introduction

Packaging materials are vital to daily life. In the context of food, they are essential for ensuring the preservation of food products over their desired shelf life and for optimizing space during handling, shipping, and storage to minimize waste [1]. Single-use plastic (SUP) is commonly used in packaging due to its outstanding preservation and protective qualities, as well as its affordability. It is a unique solution that has been given to civilization that has replaced practically all materials derived from natural resources, especially paper, when it comes to food packaging that preserves food freshness [2]. Several examples of SUP include low-density polyethylene, linear low-density polyethylene, polypropylene, cellophane, polyvinyl chloride, and poly (vinylidene chloride) [3]. Attributable to their non-biodegradable nature and persistence in the environment for over 450 years, many countries worldwide have encouraged and promoted the adoption of biodegradable packaging alternatives. These alternatives include paper, biodegradable polymers, and edible materials [2]. Additionally, numerous studies have demonstrated that the widespread use and disposal of plastics have resulted in severe pollution of both terrestrial and aquatic ecosystems, establishing plastics as a significant planetary threat [4].

The biodegradable materials have been engineered to enhance the shelf life, quality, and safety of packaged food products, while simultaneously reducing the adverse environmental impact associated with traditional plastics. Food biopolymers such as proteins and polysaccharides, along with other functional components like lipids, phospholipids, and inorganic particles, are commonly utilized in the production of biodegradable packaging [5]. This new generation of packaging exhibits superior environmental sustainability, safety, and biodegradability in contrast to traditional plastics. Furthermore, biodegradable packaging has the potential to incorporate active elements such as antimicrobials, antioxidants, and nutrients, thereby increasing functionality through the inhibition of microbial growth, reduction in lipid oxidation, and improvement of nutritional value [6]. Researchers continue to explore methods to enhance the characteristics of biocomposite materials. Numerous studies focus on extracting starch, gluten, and fibers from both wood and non-wood plants to produce these materials. The components used in biocomposite materials are sourced from diverse agricultural crops, including wheat, corn, cassava, hemp, jute, kenaf, and others [7].

Wheat, scientifically known as *Triticum aestivum*, is widely acknowledged as the predominant cereal crop cultivated across approximately 115 nations globally, primarily for human consumption [8]. According to the Food and Agriculture Organization [9], global wheat production ranged from approximately 623 million tons to 773 million tons between 2012 and 2022. This production generated about 4.5 million tons of waste biomass, making wheat a significant source of agricultural residue. Given the abundance of wheat crops, utilizing this biomass for producing sustainable biocomposite materials in food packaging offers beneficial contributions not only to food industries but to community and the environment at large. Hence, this article explores the foundational aspects of fabricating packaging materials from wheat, encompassing starch, gluten, and fiber. Due to limited recent published materials on wheat materials in fabricating food packaging, the literature review covers scientific sources from 2013 to present. Additionally, this article emphasizes recent advancements and innovations in the field of wheat-based packaging. Addressing the current research gap, it examines the potential and future trends of wheat-based materials, providing a comprehensive overview of their applications and benefits in sustainable packaging solutions.

## 2. Wheat Properties

**Figure 1:**
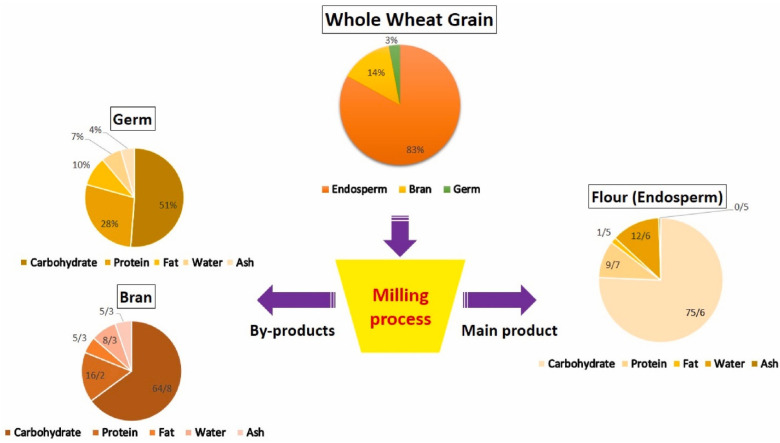
Whole wheat grain milling products and their composition (Reprinted with permission from ref. [9]).

Wheat is recognized as the world’s main grain crop and an important source of carbohydrates and proteins for human nutrition. Milling wheat grain is possibly one of the oldest manufacturing processes known to humanity [10]. The different products produced from milling whole wheat grain are shown in Figure 1, with each product’s unique components highlighted. First, wheat germ is a particularly significant by-product of the milling process, constituting 2–3% of the entire wheat grain [10].

On the other hand, one of the most popular processed cereal products consumed worldwide is wheat flour. Additionally, starch and gluten, the flour’s main macromolecular components, have a major impact on the fundamental structural and functional qualities of the grain [11,12]. Starch is the primary storing carbohydrate found in wheat that makes up between 60% and 75% of the grain and 70% and 80% of the flour. Moreover, in the essential wheat gluten industry, wheat starch is produced as a coproduct [13]. Next, 80% to 85% of the protein in wheat flour is composed of gluten proteins, which are essential to wheat dough’s ability to function effectively. These proteins are primarily composed of high-molecular-weight glutenin subunits (HMW-GSs), low-molecular-weight glutenin subunits (LMW-GSs), and gliadins (Gao et al., 2020 [12]). The HMW-GSs and LMW-GSs interact to create glutenin macropolymers (GMPs) by forming intermolecular disulfide bonds within the gluten network. This process significantly influences the structural and functional properties of the dough [12]. Table 1 presents the chemical composition of wheat products per 100 g taken from the public database of the U.S. Department of Agriculture website [14]. The composition of wheat grain can be influenced by several factors, including the genetics of the sown wheat seeds, environmental conditions, separation processes, and the degree of separation. As shown in Table 1, wheat germ is the most nutrient-dense among the wheat products listed, with the highest protein (23.2 g), fat (9.72 g), fiber (13.2 g), and mineral content (2147.3 mg). Wheat bran also has high fiber (42.8 g) and mineral content (1803.9 mg). Whole wheat flour contains moderate levels of protein (15.1 g), carbohydrates (71.2 g), and fiber, while white flours have lower protein (9.89–12 g), fat (0.97–1.66 g), and fiber (2.4–2.7 g). White flours generally have lower mineral and vitamin content compared to wheat germ and wheat bran.

Wheat germ is highly regarded because it contains high-quality plant proteins and beneficial chemicals at an economical cost for fortifying food. It offers a practical approach to enhance the nutritional value of food products and minimize waste from wheat milling [15]. Additionally, as a byproduct from producing wheat flour, wheat germ comprises about 30% protein. The primary proteins found in wheat germ are gliadin (14.0%), albumin (30.2%), globulin (18.9%), and glutenin (0.3–0.37%). It is also an excellent source of plant protein because it contains 30.2% insoluble protein and other non-protein nitrogen [16,17]. Furthermore, wheat germ is acknowledged as an affordable means of obtaining high-quality plant proteins that are on par with those found in eggs and milk, and is notable for having a high concentration of important amino acids [15,18].

It has been proven that wheat bran is an excellent source of protein, essential nutrients, and bioactive substances, such as fiber, phytosterols, antioxidants, phenolic acids, folates, non-starch polysaccharides, and vitamins, all of which have numerous health benefits [19]. It is a byproduct of refined flours and often used as animal feed or to make whole wheat flour. It could make up about 14–19% of the total wheat kernel by mass and contains 46% non-starch polysaccharides, 15–22% protein, 10–20% starch, and 4–8% lignin. In addition, arabinoxylan (AX) is the most abundant non-starch polysaccharide in wheat bran, constituting about 70% of the total. Additionally, wheat bran, maize bran, and distillers dried grains with solubles (DDGS) have potential applications as food packaging materials [20,21].

Also, wheat bran AX has the ability to be adorned with phenolic acids, particularly ferulic acid at the arabinose molecules. This process of feruloylation gives wheat bran AX useful and bioactive characteristics, including antioxidant capabilities [21]. Wheat bran contains phenolic acids that are either hydroxycinnamic acid or hydroxybenzoic acid derivatives. p-hydroxybenzoic, vanillic, syringic, and gallic acids are among the derivatives of hydroxybenzoic acid. Nevertheless, hydroxycinnamic acid derivatives such as ferulic acid, ferulic acid dehydrodimers and dehydrotrimers, and sinapic and *p*-coumaric acids are the most common phenolic acids in wheat [22]. Alternatively, one can leverage the polymeric properties of straw and bran in the production of bio-based materials through heat compression molding. This technique involves the thermo-mechanical processing of a material [23]. Research suggests that dietary fiber plays a crucial role in managing cardiovascular disease, blood cholesterol, obesity, and cancer. Wheat bran has become a popular addition to human diets because of its beneficial health properties. Furthermore, as a food supplement, wheat bran is commonly incorporated into dough, and its impact on the quality of bread is extensively researched [24].

Next, all-purpose flour is versatile and can be used for making various baked goods like noodles, cookies, and bread [25]. Enriched versions have added iron and vitamin B, while bleached flour has chlorine to improve gluten development. Moreover, bread flour has a higher protein content and is best for making bread while self-rising flour is made by adding salt and baking powder to all-purpose flour. Whole wheat flour, which includes the endosperm, germ, and bran, makes denser baked goods because the bran slows gluten development [25,26]. Due to its high gluten-forming protein content and excellent viscoelasticity, wheat flour is the top choice for making bread because the three-dimensional gluten network significantly affects dough formation and the ability to retain gas [27]. However, the process of making bread can lead to molecular and supramolecular changes, prompting research into the potential use of white bread waste for bioplastics. Baking causes starch gelatinization, resulting in some thermoplastic properties. Additionally, polysaccharides like starch and arabinoxylans may undergo depolymerization, while gluten can be denatured and aggregated. This process can also lead to new interactions, such as the possible formation of gluten-arabinoxylan aggregates through tyrosine-ferulic acid interactions [28]. Nonetheless, processed and whitened wheat flour is not the most nutritious choice as it contains high levels of carbohydrates, glycemic index, and calories. This is particularly applicable for individuals managing diabetes, hypertension, obesity, and other cardiovascular conditions [29].

Thus, wheat is acknowledged as a significant nutritional source of protein, carbohydrates, and minerals. Exploration of the by-product of wheat is essential in reducing waste and maximizing its utilization especially in the context of producing sustainable food packaging. The bioactive compounds found in wheat could be an interesting aspect for food packaging development.

## 3. Fundamental Mechanisms of Biodegradable Food Packaging

**Figure 2:**
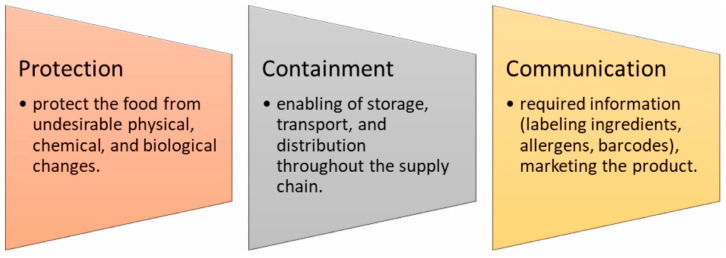
Function of packaging (Reprinted from ref. [32]).

Biodegradable plastics are known as a material that degrades by the natural activity of microorganisms. Furthermore, biodegradable plastics are produced from renewable sources and have similar mechanical and barrier properties to conventional plastics [30]. The food industry is a primary consumer of these biodegradable materials due to their effectiveness in safeguarding foods from spoilage during storage and transportation [31]. Because of their natural ability to decompose, low toxicity, and compatibility with living things, biobased materials present a practicable alternative to conventional materials. These materials have the capability to significantly diminish the ecological effects related to carbon emissions and energy consumption that are associated with standard packaging [32]. The field of research in materials is diverse and has seen significant advancement. These materials include proteins like gelatin, keratin, wheat gluten, soy protein, and whey protein isolates, as well as biobased polymers made from wood, such as cellulose, hemicellulose, starch, and lignin. All of these have been considered as environmentally friendly alternatives for conventional food packaging [33]. Furthermore, biodegradable packaging materials are designed with a specific purpose of reducing the harmful effects that plastics have on the environment while also improving the food products’ safety, quality, and shelf life. Proteins and polysaccharides are two examples of biopolymers obtained from food, and are commonly used in the packaging mentioned. They also incorporate functional additives like lipids, phospholipids, and inorganic particles [5]. Upon decomposition, biodegradable plastics primarily generate water, carbon dioxide, inorganic compounds, or biomass. This process leads to no residual waste accumulation, making it beneficial for environmental sustainability. Biodegradable plastics are mainly used in food packaging and agricultural sectors. In the food industry, packaging serves various functions, as depicted in Figure 2. It is an essential component of production, storage, distribution, preservation, and other unit operations [30].

It is essential for researchers to comprehend the fundamental mechanisms of controlled release, as it aids in the study and creation of new materials and processing methods, which is essential for advancements in the field of food packaging. Considering this, this paper offers a concise outline of the basic properties of biodegradable packaging, encompassing physical, mechanical, and barrier properties. In addition, the additive and active properties of food packaging are also briefly discussed.

Firstly, the optical characteristics of wrapping film, such as color and transparency, frequently reflect its physical characteristics. Films with a more intense color tend to exhibit reduced transparency and may become opaque. Furthermore, the thickness of the film is intricately linked to its physical properties and is influenced by the formulation of both primary and auxiliary components for the film-forming solution [34]. Additionally, optical design of packaging plays a crucial role in influencing consumer decisions. It grabs the interest of shoppers and convinces them that the product meets their requirements, especially in a shopping setting where time and mental capacity are restricted [35]. The optical properties of food packaging are crucial not only for aesthetic appeal but also for the preservation of nutrients and flavor. Effective packaging plays a vital role in safeguarding these elements from degradation caused by exposure to visible and ultraviolet (UV) light, which is essential in determining consumer satisfaction [36]. Also, light transmittance is a crucial property of food packaging, as it indicates the material’s transparency and its efficacy in blocking visible and ultraviolet (UV) light. The capacity to attenuate specific light wavelengths is vital, as exposure to such light can cause the degradation of nutrients and biologically active compounds, resulting in processes such as decomposition, oxidation, discoloration, rancidity, off-flavors, and the formation of potentially toxic elements [37].

**Figure 3:**
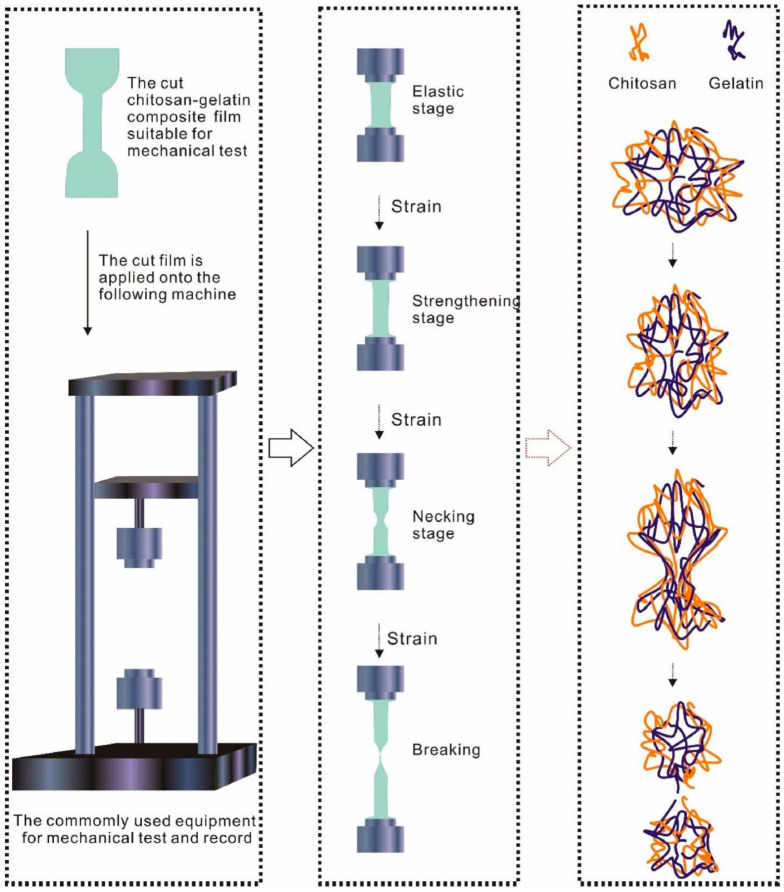
Illustration of the mechanical test and molecule-level change in chitosan-gelatin-based films (Reprinted with permission from ref. [40]).

On the other hand, during crucial stages, it is crucial that packaging materials possess good mechanical properties to effectively minimize physical damage and prevent contamination [38,39]. The mechanical characteristics of wrapping materials also plays essential role in preserving the structural stability of food products [38,40]. In addition, during the transportation and distribution phases, it is essential to recognize the critical impact they can have on food products due to the potential for mechanical damage. Hence, it is essential for a bio-based film to possess the appropriate mechanical properties and resistance to common external forces [41]. These forces, such as friction, may be encountered during the processing, shipping, handling, storage, and application of packaged food. The mechanical properties of the films play a crucial role in withstanding these forces, maintaining integrity, and preserving the quality of the packaged food. The mechanical parameters commonly used for films include tensile strength, elongation to break, Young’s modulus, and elastic modulus. Partial tests, as illustrated in Figure 3, are frequently carried out to assess these properties [41].

**Figure 4:**
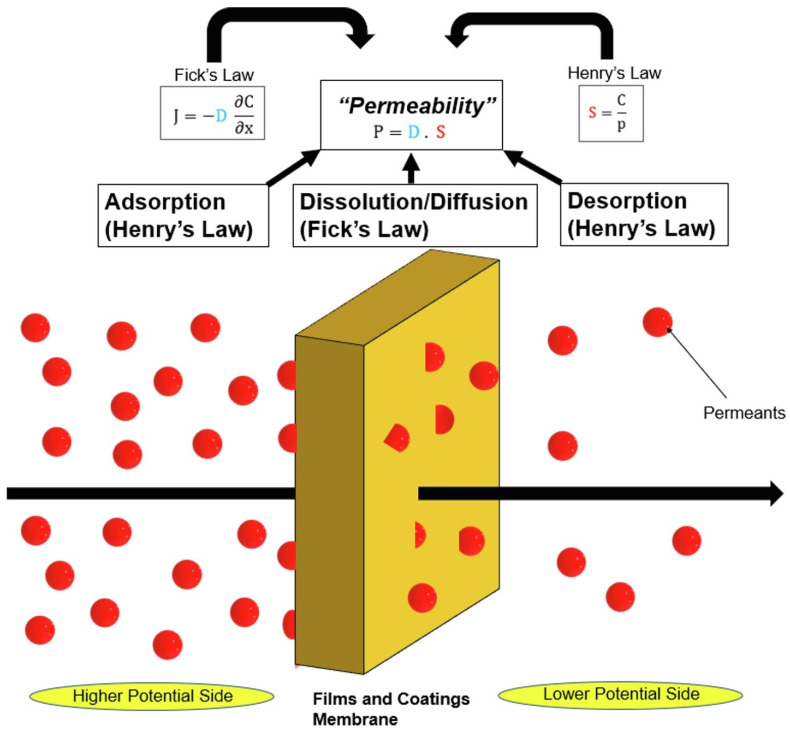
Concept of permeability for polymer films and coatings (Reprinted with permission from ref. [43]).

The ability of a material to naturally allow or restrict the flow of low molecular weight chemical substances, such as gases, water vapor, and some organic chemicals which includes fragrance molecules, is referred to as a “barrier”. This quality is essential in the food packaging sector, as maintaining the quality and safety of its contents is the main function of any packaging system [42]. The permeation of moisture and oxygen gases in polymeric materials involves the absorption, diffusion, and desorption of these gases from films or coating surfaces, as illustrated in Figure 4. The absorption of moisture or gas is influenced by the permeants’ solubility in the polymers. Essentially, the materials ability to facilitate gas passage depends on their absorptive properties, which are determined by factors such as degree of crystallinity, polarity, and molecular weight. Also, Figure 4 depicts a simplified concept of this permeation process by which the movement of water vapor through a polymer layer or membrane can also be understood as water molecules dissolving into the solid mass, traversing the material, and finally evaporating on the other side of the layer or membrane [43].

Films and coatings must possess good barrier properties to facilitate effective isolation of food products and minimize exposure to oxygen, which can lead to oxidation reactions and the growth of aerobic microorganisms. These barriers should prevent contact with water to inhibit microbial growth, water activities, and flavor alteration [44]. Poor barrier properties to water, vapor, and gases present significant challenges in packaging. Fresh produce such as vegetables and fruits require packaging in an oxygen-permeable membrane environment, while processed products require less permeability. Many producers struggle with the need for accelerated time to market. A more efficient research and development (R&D) process is essential for packaging development, aiming for a cycle of around 9 months rather than the current 12 to 18 months [45]. Additionally, the preservation of food products spoilage heavily relies on the barrier properties of packaging films, which play a crucial role in separating the food from its surroundings and preventing spoilage. Among the various barrier properties, water vapor permeability (WVP) is the most extensively studied, as it measures the amount of water that penetrates a specific area over a set period of time (g/m·s·Pa) [46]. In order to prolong the shelf life of food goods by preventing moisture transfer from the surrounding atmosphere, it is crucial to minimize the water-vapor pressure of films. WVP also offers helpful information regarding the expiration date of packaging materials. Consequently, it is essential that the coatings that have been developed prevent moisture from transferring from food products to the surrounding environment [47].

Next, the fundamental properties of pure biopolymers are not competitive enough compared to synthetic polymers, particularly for their use as films in flexible packaging. To address this challenge, one effective approach is to incorporate filler materials into biopolymer matrices to enhance their physicochemical properties [48]. The inclusion of functional fillers such as nanomaterials serves to enhance the physicochemical attributes of the films, thereby imparting them with excellent functional properties for active packaging [49]. Furthermore, active food packaging utilizes functional materials to interact with food, maintaining quality, and preventing spoilage, thus extending shelf life [50,51]. In active packaging, active components are integrated into the packaging material itself, allowing them to interact with food products. On the other hand, intelligent packaging is designed to enhance communication and marketing efforts, providing real-time information on the quality of food without direct interaction with the food itself. Intelligent packaging offers various advanced features, including oxygen sensors, microbial spoilage detectors, freshness indicators, time-temperature indicators, humidity sensors, chemical sensors, and radio frequency identification tags [51].

The development of biodegradable packaging necessitates thorough design to sustain the integrity of active ingredients and regulate their release. Due to the diminutive molecular size of many bioactive compounds, rapid release from the film matrix may occur, reducing their efficacy and consequently shortening the shelf life of the packaged food [52]. This new era of packaging is generally more environmentally friendly, safer, and biodegradable compared to conventional plastics. Additionally, biodegradable packaging can be enhanced with the incorporation of active compounds such as antioxidants, antimicrobials, and minerals. These additives serve to augment the packaging’s nutritional value, reduce lipid oxidation, and inhibit microbiological growth [6]. Thus, it is essential to carefully manage the migration and controlled release of active components during the storage and transportation of food. The retention and release of these constituents rely on the ingredients and formation of the polymer matrix. Some studies have shown that controlled release can be accomplished by using composite or multilayer devices [53].

Hence, this section highlights the food industry as a significant user of biodegradable packaging materials due to their ability to protect food from spoilage, ensuring safety, quality, and shelf life. Biodegradable packaging materials, such as proteins and polysaccharides, often include functional additives to enhance their mechanical and barrier properties. The optical, mechanical, and barrier properties of these materials are critical for preserving food quality by protecting against light, moisture, and gas permeation. Furthermore, the integration of active and intelligent packaging elements can provide additional functionality, such as extending shelf life and offering real-time information on food quality. However, the addition of functional ingredients during packaging development often reflect an additional cost of production, making it difficult to reach industrial scale production. Yet, the trend and awareness in utilizing ecofriendly materials could pull investors and overcome the cost issue.

## 4. Fabrication of Wheat Starch-Based, Gluten-Based, and Fiber-Based Coatings or Films

The fabrication of wheat starch-based, gluten-based, and fiber-based coatings or films involves utilizing the distinct properties of wheat components to create biodegradable and sustainable packaging materials. Generally, starch offers excellent film-forming abilities and mechanical strength, especially when modified physically, combined with other biopolymers, or incorporated with functional additives, making it suitable for food packaging applications [54]. Gluten, the protein found in wheat, has the potential to enhance elasticity and barrier properties, thereby contributing to the durability and effectiveness of the films [55]. Meanwhile, wheat fibers can add structural integrity and improve the overall functionality of the food packaging [56].

### 4.1. Wheat Starch-Based Coatings or Films

**Figure 5:**
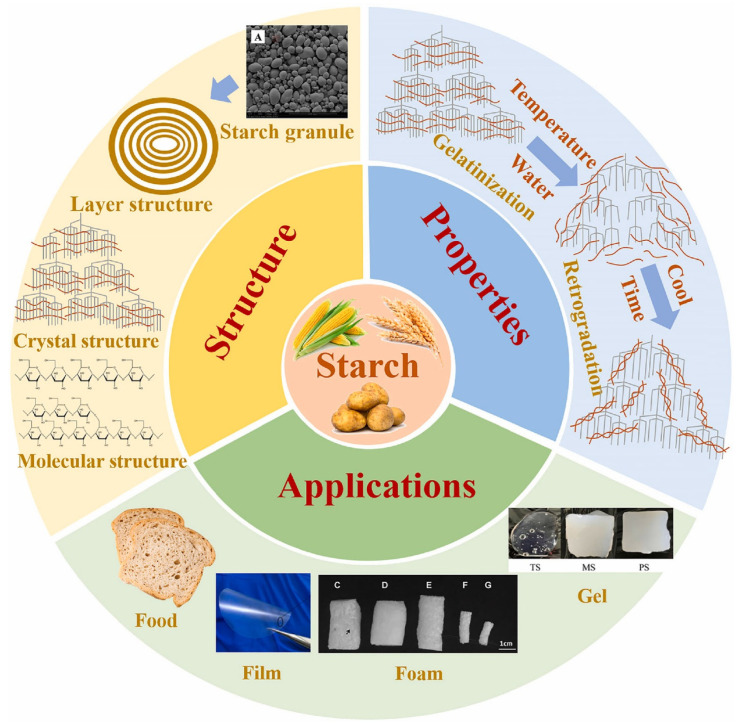
An illustration of the composition, characteristics, and uses of starch (Reprinted with permission from ref. [54]).

Chemically, starch is a polymer consisting of extensive chains of glucose molecules connected via glycosidic linkages, represented by the chemical formula C_6_H_10_O_5_)_n_. At the molecular level, starch comprises two primary polymeric components, namely amylose and amylopectin. The properties and relative proportions of these components vary based on the source [54,57]. Natural starch typically occurs in the form of granules, which can be spherical, oval, or irregular in shape (Figure 5). The diameters of these granules range from approximately 0.1 to 200 μm, varying based on their source and stage of maturation [54]. Starch properties and its interactions with water are influenced by factors such as temperature, pressure, water content, and the presence of small molecules like salt and sugar, as well as macromolecules like hydrocolloids. In starch suspensions, low temperatures cause starch granules to swell reversibly by absorbing water, but they return to their original form when dried. At higher temperatures, starch undergoes irreversible changes when gelatinization occurs with water content above 60%, while melting happens with lower water content [58,59]. Retrogradation of starch is the term for the process of starch melting, by which the structure and characteristics are strongly linked to the formation and properties of starch-based biodegradable materials [54].

In general, to create starch-based films, the starch had to be separated from the granules and then combined with plasticizer and distilled water to create a slurry that was then cast in glass or plastic and dried [7]. Although it is simple to utilize and cost-effective, the film casting approach has drawbacks. It is difficult to employ on a big commercial scale and to fully manage the homogeneity and thickness of the film [60]. Then, although the film solution casting technology is simple to use, its high water content, long drying time, and high energy consumption also make it challenging to utilize in large-scale continuous production, which raises production costs [60].

Extrusion method is another commonly utilized method for film preparation, encompassing three primary types: extrusion blowing, extrusion compression molding, and extrusion injection molding [54]. Generally, extrusion method starts by feeding a mix of film-forming materials into a screw extruder. To create raw material masterbatches in an industrial context, this combination is frequently extruded first and then chopped into particles. These masterbatches are combined and fed back into the extruder as needed for production. Inside the screw extruder, heating and shearing raise the temperature of the film-forming materials to their melting point. The molten material is then pushed through a die head, shaping it into various forms required for film production [60]. Extrusion blowing involves blowing, pressing, and cooling the material to form films. Extrusion compression molding uses hot pressing and cooling to shape the material into films. Extrusion injection molding is similar but involves injecting the molten material into molds and cooling it to produce complex plastic products at a lower cost, making it widely used in polymer processing [60].

Hence, there are various methods in producing starch-based films with its advantages and disadvantages. It is important to understand the molecular characteristics of wheat starch for consideration of adding functional compounds. However, reports on starch-based films’ comparison to the synthetic polymer are still scarce. Next, in the context of cost, current production cost is likely high due to its research stage and lack of investors to make it into the industrial stage.

### 4.2. Wheat Gluten-Based Coatings and Films

The stored protein called wheat gluten is present in the wheat grain endosperm and is what gives wheat-based foods their dough-forming and structural stability. It is the left over after wheat flour has been stripped of its starch for which gliadin and glutenin are the two primary protein components that make up wheat gluten [61,62]. Glutenin proteins play a crucial role in enhancing the cohesion and elasticity of dough, primarily through noncovalent bonds like hydrogen bonding, further stabilized by additional noncovalent and covalent interactions. The viscoelastic properties of wheat gluten facilitate the creation of intricate three-dimensional networks in wheat-based food products such as bread. Various factors known to influence gluten functionality include temperature, pH levels, chemical additives such as oxidizing or reducing agents, and enzymes like peptidases and transglutaminase [61]. Furthermore, in dry conditions, wheat gluten exhibits effective barriers against oxygen and carbon dioxide, thereby regulating the inherent gas permeability of cellulosic materials [55].

**Figure 6:**
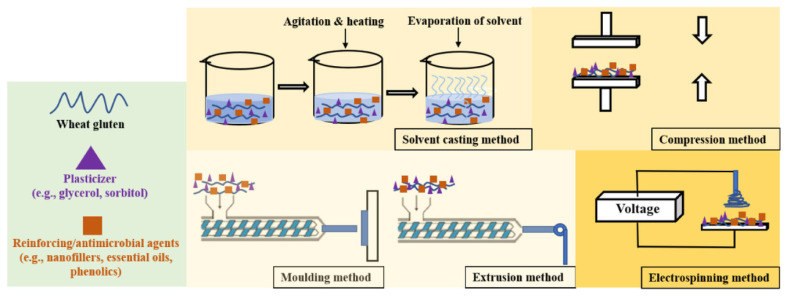
Different methods for producing wheat gluten-based coatings and films (Reprinted with permission from ref. [61]).

Coatings and films based on wheat gluten are typically produced using methods such as solvent casting, compression molding, extrusion, electrospinning, or a combination thereof, as depicted in Figure 6. Additionally, plasticizers i.e., glycerol, sorbitol, diethanolamine, and triethanolamine are commonly added to enhance the flexibility of films. However, their inclusion typically results in decreased tensile strength and increased water vapor permeability of the films [61]. The solvent casting method for producing wheat gluten-based films is influenced by several variables, including pH, solvent type, heat treatment, and solvent evaporation. These factors impact the properties and quality of the films. Moisture content also plays a significant role, influencing the glass transition temperature of wheat gluten and subsequently affecting the elongation at break and brittleness of the films. Additionally, the drying temperature and relative humidity (RH) can impact the tensile strength of the films [61].

Compression and molding techniques are widely used in the production of wheat gluten-based films, especially those that contain hydrophobic materials that are not well soluble in water [63]. The properties of films made using compression and molding techniques can be influenced by factors such as temperature and duration of molding. This is because heat and temperature can affect the glass transition temperature of wheat gluten, particularly when water or plasticizers are included [64]. However, the temperature during compression or molding has a greater impact compared to the duration of compression or molding. This is due to the crucial role of temperature in breaking down existing disulfide bonds and forming new ones during heat treatment, which ultimately affects the extent of cross-linking in gluten networks [61,65].

Next, on a method of fabrication of wheat-based film, extrusion processing incorporates heat, shear, and pressure, which significantly impact the molecular dynamics of wheat gluten. Heat and shear during extrusion lead to the denaturation of wheat gluten and the disruption of its native hydrophobic and disulfide bonds, thereby affecting the thermal and mechanical characteristics of resulting films [66]. On the other hand, electrospinning is a popular technique for producing wheat gluten films which can be infused with nanofillers for food packaging purposes. In this process, a polymer solution is subjected to high voltage from a needle tip. The electric field surpasses surface tension, causing the charged solution to evaporate, then the elongated polymer droplets on the collector solidify into films [67,68].

Thus, gluten in wheat significantly contributes to the mechanical properties of biodegradable films, and it is produced through various methods. Similar to the fabrication of starch-based films, there remains a scarcity of studies comparing gluten-based films with synthetic or other biodegradable films. The comparison could lead to deeper understanding in applications of functional properties such as nanofillers that could enhance the gluten-based films.

### 4.3. Wheat Fiber as Base/Additives for Coatings and Films

Wheat plants are a good source of fiber in a variety of forms; most of the fiber is taken from the bran, straw, and wheat husk. Wheat fiber is a dietary fiber derived from the wheat plant. It undergoes a unique thermo-physical process, followed by milling, sieving, and standardization into specific grades for various applications. Wheat fiber typically appears as a fibrous, odorless powder ranging from white to light beige in color [7]. Additionally, wheat bran contains several beneficial components such as phenolic compounds, starches, soluble and insoluble dietary fibers, and proteins. The water-insoluble fiber components in bran, such as cellulose, hemicellulose, and lignin, provide significant advantages in biocomposite materials due to their inherent strength. Moreover, microfibers derived from grain by-products are priced at half the cost of wood microfibers and are easier to procure [69]. Next, films containing wheat fiber extract have great potential in enhancing biodegradable film as in its mechanical properties and biodegradation [70]. In addition, the extracts from wheat fiber also have the potential to enhance film thermal stability and reduce moisture content of the biocomposite films [71].

Wheat straw is a by-product of wheat cultivation and a product of wheat fiber, characterized by its low moisture content and high lignocellulosic content, which slow down its degradation. Due to these properties, wheat straw and its derivatives are utilized in food packaging as potential filler materials [72]. However, when used as filler materials in developing green biocomposites, wheat straw faces challenges related to interfacial adhesion and dispersion properties. The hydrophilic nature of wheat straw fibers and the hydrophobic nature of the polymeric matrix hinder the performance as a filler material in composite-based packaging [73]. Additionally, valorizing wheat straw to extract cellulose is a favorable method for utilizing agricultural waste; with wheat straw comprising 30% cellulose, 22% hemicellulose, and 17% lignin, it serves as a viable source of cellulose [74]. Additionally, nanocellulosic materials are fabricated from wheat straw to create nanocomposite-based packaging. Cellulose nanofibers, extracted from wheat straw using a chemical-mechanical technique, are employed as nanofillers in developing nanocomposites with thermoplastic starch [56].

As wheat fibers are rich in cellulose, hemicellulose, and lignin, enhancing the mechanical properties and biodegradability of films highlights the environmental advantages of using agricultural by-products. Additionally, it provides valuable economic context by comparing the cost-effectiveness of wheat-derived microfibers to wood microfibers, making them more appealing for industrial applications. However, reports on chemical interactions of wheat fibers with the films are still scarce as researchers focus more on its physical properties.

## 5. Applications and Innovation of Wheat-Based Materials for Food Packaging

Wheat-based materials are highly versatile and extensively used in food packaging, serving purposes ranging from edible coatings to the production of robust structural films. These materials effectively preserve the quality of various fruits, reduce moisture content, and provide antimicrobial protection. Table 2 summarizes various recent studies on the wheat-based materials for food packaging, detailing their applications, types of packaging, key findings, and references.

Starting with a recent study by Ciaramitaro et al. [75] that explored starch films by adding citric acid (CA) or sodium citrate (SC) as cross-linkers and polyethylene glycol 200 (PEG200) as a plasticizer, the films were obtained through solvent casting. The study indicates that proper amounts of CA, SC, and PEG200 improve the properties of the starch films and inhibit the retrogradation and recrystallization of starch chains. The ss-NMR analysis revealed significant changes in the chemical neighborhood of nuclei and the mobility of polymer chains, indicating a different structural organization of the polymer chains which leads to higher resistance to thermal degradation and better elongation at break. However, films with SC as a cross-linker showed lower mechanical properties compared to those with CA. Then, another study on wheat starch film by Yousefi et al. [76] incorporated sodium montmorillonite (Na-MMT) and titanium dioxide (TiO_2_) nanoparticles (Nps) into starch films. TiO_2_ Nps effectively blocked UV light, with the film containing 4% TiO_2_ removing more than 99% of UV radiation. The addition of nanomaterials enhanced the mechanical properties of films, although it reduced elongation at break. Scanning electron microscopy (SEM) micrographs revealed that the nanomaterials were appropriately dispersed across the film’s surface, especially at lower concentrations. Thermogravimetric analysis (TGA) results indicated that the addition of nanomaterials, particularly TiO_2_, significantly improved the thermal stability of the nanocomposite films. Then, Punia et al. [77] investigated edible films for grapes made from native and OSA-modified starch. The author stated that their starch-based coatings maintained total phenolic content, total carotenoids, scavenging activities, and the shelf life and quality of grapes for up to 13 days. The study suggests that edible coatings with good water barrier properties, particularly those based on OSA-modified starch, can significantly benefit fruit processors by enhancing the shelf-life of fresh produce. Next, Bonilla et al. [78] in their study demonstrate their enhanced starch films by incorporating chitosan, which significantly increased their mechanical properties. Additionally, these films exhibited increased water vapor and oxygen permeability and demonstrated antimicrobial activity.

On the other hand, Leiva et al. [79] in their study added essential oils of cinnamon and turmeric to gluten and starch films. The study tested the films’ effectiveness in preserving carrots by evaluating weight loss, appearance, and fungal growth. A significant decrease in weight loss was observed for carrots coated with starch-based films (up to 44.44%) and gluten-based films (up to 43.33%) with the addition of turmeric essential oil. This indicates improved moisture retention and reduced dehydration of the carrots. Sharma et al. [80] in their study examine the effectiveness of gluten films incorporated with montmorillonite (MMT) and starch nanocrystals (SNCs), further enriched with chitosan, in preserving the quality of litchis over a 20-day storage period at 4 °C. The films exhibited improved mechanical and barrier properties, with SNCs showing a better preservation effect than MMT, by which litchis wrapped in these nanocomposite films exhibit superior shelf stability compared to untreated litchis, which experience significant deterioration. Among the film variants, SNCs/chitosan films (GSL2) outperform MMT/chitosan films (GML2) due to their superior barrier properties.

Then, a study by Rovera et al. [55] addresses the increasing demand for environmentally friendly solutions by investigating the benefits of applying a wheat gluten coating on paperboard substrates intended for food packaging applications. SEM revealed that the surfaces of the coatings were very smooth; however, the films exhibited structural defects like pinholes and increased brittleness with higher silica content. Next, Nataraj et al. [81] used citric acid and glutaraldehyde as cross-linking agents combined with banana fibers to develop sustainable biofilms with improved mechanical properties and reduced water absorption. These cross-linked films showed reduced water absorption and enhanced mechanical properties, with citric acid cross-linked films exhibiting slightly higher strength. A similar study on wheat gluten film by Fabra et al. [82] evaluates the influence of processing conditions, electrospun coating thickness, and aging on the barrier, mechanical, morphological, and optical properties of materials, specifically focusing on thermoplastic wheat gluten films coated with annealed polyhydroxybutyrate (PHB) electrospun fibers. The authors highlight that electrospinning was essential to achieve good adhesion between the layers, which was a key factor in the enhanced barrier properties. Also, the multilayer systems exhibited improved mechanical performance.

Next, in the study by Gumber et al. [71], they created arabinoxylan acetate (AXAc) composite films with nanocellulose (NC) extracted from straw enhanced with silver nanoparticles (AgNPs) for improved functional properties, particularly antimicrobial activity. Films with AXAc showed enhanced functional properties, including improved thermal stability and reduced moisture content by 1.3–1.4 times compared to pure NC films. A similar study by Wu et al. [83] developed films filled with reduced graphene oxide (rGO), prepared through vacuum filtration. The addition of rGO significantly enhanced the tensile properties of the films, of which 8% rGO content showed a remarkable 639.8% increase in tensile strength, achieving values as high as 88.70 MPa. Also, the incorporation of rGO reduced the hydrophilicity, water vapor permeability, and swelling ratio of the films. Specifically, the contact angle doubled in the 10% rGO-Film compared to the 0% rGO-Film, indicating increased hydrophobicity. Water vapor permeability was also decreased by 10.7%. In addition, Ahmed et al. [70] utilized calcium ions (Ca^2+^) to cross-link lignocellulosic residue fibers isolated from wheat straw. Increasing concentrations of Ca^2+^ ions (up to 800 mM of CaCl_2_) led to notable reductions in moisture content, water solubility, water vapor permeability, transparency, and elongation of the films. Additionally, tensile strength reached 6.61 ± 0.07 MPa with 800 mM CaCl_2_, marking a substantial enhancement by 2.5 higher compared to commercial polyethylene films.

Thus, this section reveals recent research has explored into the utilization of wheat-based materials in food packaging, highlighting their adaptability and efficacy in preserving food quality, minimizing moisture, and offering antimicrobial protection. Also, the use of additives like citric acid, TiO_2_ Nps, chitosan, and essential oils to improve the mechanical strength, barrier properties, and antimicrobial activity of wheat-based films is explored. While these advancements show potential, there are drawbacks such as decreased mechanical properties in certain formulations and the presence of structural flaws in specific coatings. Furthermore, while various studies indicate improved properties in lab settings, factors such as production costs, scalability, and industrial applicability are crucial for real-world adoption.

## 6. Potential and Future Trends

Upon previous discussion, the use of wheat fiber, gluten, and starch as food packaging materials represents a promising opportunity towards sustainable packaging solutions. The future trends in utilizing wheat fiber, gluten, and starch in food packaging are likely to focus on optimizing their properties further. Researchers are exploring ways to enhance the mechanical strength, barrier properties, and shelf-life stability of these materials. Additionally, efforts are underway to scale up production processes and ensure cost competitiveness compared to conventional plastics. Next, the regulatory landscape and consumer preferences are also shaping the future of sustainable packaging. As regulatory measures concerning the use and disposal of plastics become increasingly stringent on a global scale, there is a mounting need for sustainable alternatives. Consumers are increasingly mindful of environmental impact and are supportive of products that offer sustainability credentials. Moreover, advancements in technology and material science are driving advances in wheat-based packaging which includes improvements in biopolymer blending, nanotechnology applications for barrier enhancement, and novel processing techniques to achieve desirable packaging characteristics.

Additionally, the use of wheat fiber, gluten, and starch in food packaging aligns closely with several Sustainable Development Goals (SDGs), highlighting their potential to contribute to global sustainability efforts. Wheat-based packaging promotes sustainable consumption and production patterns by offering a renewable alternative to fossil fuel-derived plastics. These biodegradable materials reduce environmental impact across their lifecycles, aligning with the SDG 12 goal of responsible consumption and production. By minimizing reliance on non-renewable resources and reducing waste generation, wheat fiber, gluten, and starch-based packaging contribute to sustainable practices in global supply chains. Then, while indirectly related to health, reducing plastic pollution through sustainable packaging positively impacts human well-being. By mitigating environmental pollutants and supporting healthy ecosystems, wheat-based materials contribute to the SDG 3 goal of promoting good health and well-being. Preserving ecosystems that provide essential services, such as clean air, water, and food, supports human health and quality of life, underscoring the interconnectedness of environmental sustainability and human well-being. Also, replacing traditional plastics with wheat-based materials supports climate action by mitigating greenhouse gas emissions. These biodegradable alternatives help reduce the carbon footprint associated with plastic production and disposal. By fostering climate resilience and adaptation, wheat-based packaging aligns with the SDG 13 objectives to combat climate change and its impacts, contributing to a more sustainable and environmentally friendly future.

## 7. Challenges of Utilizing Wheat-Based Materials for Food Packaging

This section highlights the challenges of utilizing wheat-based materials for food packaging based on the previous discussion. Although the use of wheat fiber, gluten, and starch as food packaging materials holds promise for sustainability, several challenges must be addressed to maximize their effectiveness. Firstly, wheat-based materials typically exhibit lower mechanical strength and durability compared to traditional plastics, posing challenges for their use in packaging and transportation [75,80]. Enhancing these properties without compromising biodegradability is crucial to ensure that wheat-based materials can withstand the restrictions of commercial packaging applications. Focusing on starch and gluten-based films, it demonstrates limited moisture barrier properties [77,80,81], which can impact the shelf-life of packaged goods, particularly for moisture-sensitive products. Addressing this challenge requires advancements in coatings or additives that can improve moisture resistance while maintaining the biodegradability of wheat-based materials. Enhancing their ability to protect contents from environmental moisture will broaden their applicability in food packaging.

Then, in food safety and regulation aspects, ensuring compliance with food safety regulations is paramount for wheat-based packaging materials. Issues such as potential allergens and contaminants must be strictly addressed to uphold consumer safety and regulatory standards. Convincing testing and certification processes are essential to guaranteeing the suitability of wheat-based materials for direct food contact applications and ensuring consumer confidence in their safety and reliability. Furthermore, educating consumers about the benefits of wheat-based packaging and addressing potential misconceptions or resistance is pivotal for market acceptance. Consumer perceptions and awareness significantly influence the adoption of sustainable packaging alternatives. Effective communication strategies highlighting the environmental advantages and safety of wheat-based materials can enhance consumer trust and foster widespread adoption in the marketplace.

On the other hand, the production of materials derived from wheat carries a higher cost in comparison to petroleum-based plastics, creating a substantial impediment to their widespread adoption. Several factors contribute to this increased cost, including the procurement of raw materials, the technological processes involved, and the current inability to achieve significant economies of scale with wheat-based alternatives. Overcoming these cost-related challenges is essential for establishing wheat-based materials as a more competitive choice in the marketplace.

## 8. Conclusions

Wheat, the most widely cultivated cereal, provides an abundant source for biocomposite materials with its materials holding significant promise for sustainable food packaging solutions. It aligns with global sustainability goals, reducing reliance on non-renewable resources and minimizing environmental impact. Recent studies demonstrate wheat-based materials’ effectiveness in preserving food quality, reducing moisture content, and providing antimicrobial protection. Innovations include cross-linking agents, nanofillers, and essential oils. Other studies focus on integrating biodegradable components like wheat gluten with paperboard substrates and using cross-linking agents to develop sustainable biofilms. The results demonstrate that wheat-based packaging can significantly enhance food preservation, offering an environmentally friendly alternative to traditional plastics.

Furthermore, the use of wheat fiber, gluten, and starch as food packaging materials shows promise for sustainable packaging solutions. Future trends are likely to focus on improving the mechanical strength, barrier properties, and shelf-life stability of these materials, as well as scaling up production to achieve cost competitiveness with conventional plastics. Increasing regulatory pressure on plastic use and disposal, coupled with growing consumer demand for environmentally friendly products, is driving the adoption of sustainable packaging. Technological advancements, including biopolymer blending, nanotechnology, and novel processing techniques, are further enhancing the properties of wheat-based packaging. These materials support several Sustainable Development Goals (SDGs) by promoting responsible consumption, reducing environmental impact, and supporting good health and well-being. Additionally, by offering a renewable, biodegradable alternative to fossil fuel-derived plastics, wheat-based packaging supports climate action and contributes to a more sustainable and environmentally friendly future.

While wheat-based materials like wheat fiber, gluten, and starch show promise for sustainable food packaging, several challenges must be addressed. These materials often lack the mechanical strength and durability of traditional plastics, which limits their use in packaging and transportation. They also have limited moisture barrier properties, impacting the shelf life of moisture-sensitive products. Improving these aspects without compromising biodegradability is essential. Ensuring food safety by addressing potential allergens and contaminants and complying with regulatory standards is also crucial. Moreover, higher production costs compared to petroleum-based plastics pose a significant barrier to their widespread adoption. Educating consumers about the environmental benefits and safety of wheat-based packaging can enhance market acceptance, but overcoming these cost-related challenges is key to making wheat-based materials a competitive alternative in the marketplace.

## Figures and Tables

**Table 1:** Composition of wheat products per 100 g.

Nutrition	Wheat Germ	Wheat Bran	Whole Wheat Flour	White Flour (Plain)	White Flour (Self-Raising)	White Flour (Bread Making)
Protein (g)	23.2	15.6	15.1	10.3	9.89	12
Fat (g)	9.72	4.25	2.73	0.98	0.97	1.66
Carbohydrates (g)	51.8	64.5	71.2	76.3	74.2	72.5
Total sugars (g)	n.d.	0.41	n.d.	0.27	0.22	0.31
Fiber (g)	13.2	42.8	n.d.	2.7	2.7	2.4
Minerals (mg)	2147.3	1803.9	622.2	290.59	2306.80	281.42
Vitamin E (mg)	n.d.	1.49	n.d.	0.06	0.05	0.4
Vitamin B (mg)	n.d.	n.d.	0.268	n.d.	n.d.	n.d.

n.d.: not defined.

**Table 2:** Recent study of wheat-based materials for food packaging.

Wheat Material	Food Application	Packaging Type	Key Findings	Reference
Starch	n.d.	Starch film	- Films modified by citric acid and sodium citrate as cross-linker and polyethylene glycol (PEG200) as plasticizer. - The cross-linker binds effectively with each other. - Cross-linker films show good stability, which implies great flexibility. - Films with sodium citrate as cross-linker show lower mechanical properties compared to films with citric acid as cross-linker.	[75]
Starch	n.d.	Starch film	- Incorporated with sodium montmorillonite and titanium dioxide nanoparticles (Nps). - Nps-incorporated film reduces in water vapor permeability, water solubility, and moisture content. - Increase in density of film which is influenced by titanium dioxide Nps. - Films with titanium dioxide show better thermal stability.	[76]
Starch	Grapes	Edible films	- Both native and OSA-modified starch coatings demonstrated minimal degradation in the total phenolic content, carotenoid content, and antioxidant activity of grapes. - OSA-modified starch coating was found to be the most effective in maintaining the quality of grapes, efficiently preserving their firmness and minimizing weight loss.	[77]
Starch	n.d.	Starch-chitosan film	- Incorporation of chitosan into films has significantly increased mechanical properties of starch films. - Water vapor permeability and oxygen permeability also increase for films with chitosan blend. - Presence of chitosan shows antimicrobial activity by assessing coliform counts.	[78]
Gluten and Starch	Carrot	Gluten and starch film	- Incorporated with essential oil of cinnamon and turmeric. - Enhance thermal and antioxidant properties of films. - Films with turmeric EO have better antioxidant properties. - Films with turmeric EO show better preservation of carrot by observing the moisture retention.	[79]
Gluten	Litchis	Films	- Gluten films enhanced with montmorillonite (MMT), starch nanocrystals (SNCs). - Mechanical and barrier properties of films enhanced with MMT and SNCs as fillers. - SNCs-filled films show better preservation effect than MMT-filled films.	[80]
Gluten	n.d.	Coatings	- A hybrid coating formulated with silica. - Thermal stability of coating increases compared to control sample. - Morphology study shows surfaces were smooth but with pin holes, cracks, fractures, and void. - Coatings brittleness increase with as amount of silica increase.	[55]
Gluten	n.d.	Film	- Cross-linker of gluten film–banana fibers with citric acid and glutaraldehyde (Gtld). - Cross-linking films shows reduction of water absorption. - Mechanical properties were enhanced for cross-linked films. - Citric acid cross-linked films exhibited slightly higher strength compared to Gtld cross-linked films.	[81]
Gluten	n.d.	Gluten film	- Film mixed with polyhydroxybutyrate (PHB) electrospun fibers. - Films with PHB enhance barrier and mechanical properties.	[82]
Fiber	n.d.	AXAc film	- Cellulose extracted from straw then converted into NC. - NC enhances thermal stability and moisture content of arabinoxylan acetate (AXAc) composite films.	[71]
Fiber	n.d.	Nanoholocellulose film	- Film filled with reduced graphene oxide (rGO) enhanced the mechanical and barrier properties of films. - Thermal stability and electrical conductivity were also enhanced with rGO as film filler.	[83]
Fiber	n.d.	Cellulose film	- Lignocellulosic residue isolated from wheat straw to prepare biodegradable films. - Calcium ions (Ca^2+^) are used to cross-link the fibers. - Barrier and mechanical properties increase as concentration of Ca^2+^ increases. - Mechanical properties enhanced by 2.5 times compared to commercial plastic films.	[70]

n.d.: not defined.

## Data Availability

No new data were created or analyzed in this study. Data sharing is not applicable to this article.
